# Pharmacist-managed clinics for patient education and counseling in Japan: current status and future perspectives

**DOI:** 10.1186/s40780-014-0001-4

**Published:** 2015-01-28

**Authors:** Kiyofumi Yamada, Toshitaka Nabeshima

**Affiliations:** Department of Neuropsychopharmacology and Hospital Pharmacy, Nagoya University Graduate School of Medicine, 65 Tsuruma-cho, Showa-ku, Nagoya, 466-8560 Japan; Department of Regional Pharmaceutical Care and Sciences, Meijo University School of Pharmacy, Nagoya, 468-8503 Japan; Japanese Drug Organization of Appropriate Use and Research, Nagoya, 468-0069 Japan

**Keywords:** Pharmacist-managed clinic, Pharmaceutical care, Adherence, Patient education, Anticoagulation, Asthma, COPD, Alzheimer’s disease, Cancer chemotherapy

## Abstract

To improve the adherence to and knowledge about pharmacotherapy in outpatients and to maximize the efficacy and minimize the adverse drug events, the first pharmacist-managed clinic (PMC) in Japan was established for anticoagulation therapy at Nagoya University Hospital in 2000. Since then, various PMCs such as for asthma/chronic obstructive pulmonary disease, Alzheimer’s disease, hypercholesterolemia, chronic hepatitis C, cancer chemotherapy, palliative care, chronic kidney disease, and continuous ambulatory peritoneal dialysis have been established and expanded to many hospitals in Japan. Accumulating evidences suggest that PMCs have some beneficial effects on patients’ adherence to and knowledge about their pharmacotherapy as well as the clinical outcome, besides being cost-effective. Notably, PMCs for cancer chemotherapy have been approved as a new medical service in hospitals in 2014, which is covered by the universal health coverage in Japan. In this review article, the current status of PMCs for patient education and counseling in Japan and their impact on pharmaceutical care and management are critically reviewed. Furthermore, future perspectives on PMCs are discussed.

## Introduction

Pharmacists can contribute to positive outcomes of pharmacotherapy by educating and counseling patients to prepare and motivate them to follow their pharmacotherapeutic regimens and monitoring plans. Education and counseling are most effective when conducted in a room or space that ensures privacy and the opportunity to engage in confidential communication [American Society of Health-System Pharmacists (ASHP) guidelines on pharmacist-conducted patient education and counseling] [1]. Reinders and Steinke, for instance, reported the development, operation, patient management protocol, and teaching activities of a pharmacist-managed anticoagulation clinic for ambulatory patients in 1979. They concluded that this clinic gives the pharmacist a unique opportunity to provide comprehensive pharmaceutical services, to establish effective, long-term professional relationships with ambulant patients and their families, and to foster interdisciplinary health team activities [2]. To date, various pharmacist-managed clinics (PMCs) for chronic diseases or symptoms in different clinical settings have been reported in the United States [3-22] and other countries [23-28]. These include clinics undertaking education and counseling for anticoagulation [2,23,25-27], asthma [3], *Helicobacter pylori* infection [4], diabetes [7], hyperlipidemia [8,9], hypertension [10,11], latent tuberculosis infection [12], pain [13,14], smoking cessation [15], and cancer chemotherapy [21,22]. Beneficial effects of PMCs have been repeatedly reported in terms of cost-effectiveness, patients’ adherence to and knowledge about pharmacotherapy, and the outcome of treatment [29-34].

## Review

### Pharmacist-managed clinics in Japan

An international exchange program for hospital pharmacists between Japan and the United States and other countries under a research program entitled “A role of hospital pharmacists in appropriate drug use” (PI: Toshitaka Nabeshima), which was supported by a Health Labour Science Research Grant (H10-Iyaku-068, 1998–2000) from the Ministry of Health, Labour, and Welfare of Japan, played an indispensable role in the introduction and development of pharmacist-managed clinics (PMCs) for patient education and counseling in Japan. In this 3-year program, 10 Japanese pharmacists studied abroad to see clinical pharmacy practices in the United States, and 8 visiting clinical pharmacists from foreign countries introduced PMCs and other pharmacy services, as well as pharmacy education, to Japan [35,36].

The first PMC in Japan was established for anticoagulation therapy with warfarin at Nagoya University Hospital in 2000 [37-39]. This was triggered by a request from a medical doctor in the vascular surgery department for hospital pharmacists to conduct patient education and pharmaceutical counseling for those who had taken warfarin for a long period, but for whom the prothrombin time-international normalized ratio (PT-INR) value was unstable and sometimes outside of the target range. A hospital pharmacist, Dr. Keiko Yamamura, played an indispensable role in establishing this PMC. Implementation of this new clinical practice involving patient education and counseling was first reported in the 10^th^ Clinical Pharmacy Symposium in Japan (Chiba, Japan, 2002). A PMC for asthma was also started at Nagoya University Hospital in 2001, for which a pharmacist, Dr. Masaya Hasegawa, played a crucial role [39,40]. At the same time, a PMC for asthma was introduced by Dr. Ritsuko Taniguchi into pharmacy education at Okayama University School of Pharmacy [41]. Drs. Yamamura, Hasegawa, and Taniguchi had all studied abroad with support from the international exchange program mentioned above [35].

At present, various PMCs are in operation at hospital pharmacies in Japan [42-50] (Table 1). For example, we now operate 7 PMCs at Nagoya University Hospital, such as for anticoagulation therapy, asthma/chronic obstructive pulmonary disease (COPD), donepezil outpatient consultation service (DOCS), palliative care, chronic kidney disease, molecular-targeted drugs, and continuous ambulatory peritoneal dialysis. Some of them are run in collaboration with schools of pharmacy. A PMC for DOCS is an example of such hospital pharmacist/faculty pharmacist collaboration. The DOCS provides pharmaceutical education and counseling about the pathophysiology of Alzheimer’s disease and drug therapy with donepezil and other drugs to outpatients and their family members [44]. We have demonstrated that the DOCS improves understanding of the clinical features of Alzheimer’s disease in patients and provides pharmacological knowledge about antidementia drugs, leading to significantly better adherence to pharmacotherapy with donepezil [44].

**Table 1:** **Examples of PMCs at hospital pharmacies in Japan**

**PMCs**	**References**
Anticoagulation therapy	[37-39]
Asthma/chronic obstructive pulmonary disease (COPD)	[39-41,69-71]
Donepezil outpatient consultation service (DOCS)	[39,44]
Cancer chemotherapy	[45-50]
Chronic kidney disease	not available
Continuous ambulatory peritoneal dialysis	not available
Hypercholesterolemia	[42]
Chronic hepatitis C	[43]

As a distinct feature of PMCs in Japan, the number of PMCs for cancer chemotherapy has rapidly increased in parallel with a paradigm shift of the treatment from inpatients to outpatients [45-50]. Of note, PMCs for cancer chemotherapy have been approved as a new medical service in hospitals in 2014, and are covered by the universal coverage in Japan, if certified oncology pharmacists, in agreement with physicians, conduct patient education and counseling about cancer chemotherapy with the consent of patients.

### PMCs for anticoagulation therapy

Oral anticoagulation therapy with warfarin is beneficial for the primary and secondary prevention of life-threatening thromboembolic events. Warfarin exhibits anticoagulant actions and prophylactic effects on thrombosis by inhibiting the synthesis of vitamin K-dependent coagulation factors. It is useful for a variety of conditions including venous thromboembolism, atrial fibrillation, mechanical prosthetic heart valves, coronary artery disease, and stroke [51,52]. There are some limitations, however, as to its safe and effective use. A narrow therapeutic index, hemorrhagic side effects, variations in dosage requirements, adherence, and multiple drug-drug and drug-food interactions all limit its clinical effectiveness or enhance its toxicity. In particular, the risk of major bleeding on warfarin is approximately 1-5% per year, and bleeding complications due to anticoagulants are among the most frequent adverse drug effects (ADEs) [53]. Accordingly, management, education, and counseling for anticoagulation therapy in outpatients are necessary and important for appropriate pharmacotherapy [54].

It has been reported that all hemorrhagic and thromboembolic events associated with warfarin were significantly reduced by a PMC in the University of Florida’s Family Practice [32]. Furthermore, by avoiding hospitalization and emergency room visits due to thromboembolic or hemorrhagic complications, potential cost avoidance was estimated if the control group had been followed by the anticoagulation monitoring service [32]. A retrospective assessment comparing a pharmacist-managed anticoagulation clinic with physician management was recently reported using INR stability. The study demonstrated that the pharmacist-managed anticoagulation clinic had higher rates of INRs determined to be therapeutic and exhibited significantly less variability in therapeutic INR rates relative to a physician-managed service [33].

A cross-sectional pre- to post-counseling study was conducted at Nagoya University Hospital to assess the effects of a PMC for anticoagulation therapy on patients’ knowledge and the quality of the treatment [38]. This PMC significantly improved the patients’ knowledge about warfarin treatment and reduced the mean deviation from the PT-INR target range [37,38].

The development of reliable and accurate devices to measure PT-INR, such as Coaguchek, Pro Time Microcoagulation System, and Coumatrak monitor, has allowed self-testing by patients at home. A systematic review and meta-analysis of individual patient data showed that self-monitoring and self-management of oral anticoagulation are a safe option for suitable patients of all ages [55]. We have recently started clinical research to assess the safety and effectiveness of internet-based PMC for anticoagulation therapy with warfarin in combination with self-monitoring of PT-INR in patients who may have potential adverse drug interactions with warfarin. For example, both fluorouracil and miconazole are known to exhibit drug-drug interactions with warfarin, leading to potential side effects [56,57]. Accordingly, we applied the self-management of warfarin treatment supported by an internet-based PMC in combination with self-monitoring of PT-INR to 2 patients receiving concomitant treatment with either FOLFIRI chemotherapy [58] or miconazole gel [59]. By using the time in the therapeutic range as a measure of the quality of treatment [60], we reported that the internet-based PMC in combination with self-monitoring of PT-INR may be safe and effective for pharmacotherapy management of anticoagulation therapy in high-risk patients with potential drug-drug interactions [58,59].

In addition to warfarin, novel oral anticoagulants such as Factor Xa inhibitors, rivaroxaban, apixaban, and edoxaban, as well as a direct thrombin inhibitor, dabigatran etexilate, have recently become available for anticoagulation therapy [52]. Because these new drugs also have some risks for bleeding and other ADEs [61,62], we have included pharmaceutical education and counseling on novel oral anticoagulants in the PMC at Nagoya University Hospital.

### PMCs for asthma/COPD

Asthma and COPD are pulmonary disorders characterized by various degrees of airflow limitation, inflammation, and tissue remodeling. There are clear differences between these two diseases; the former originates in childhood and is best treated with anti-inflammatory steroids, whereas the latter occurs in adults who smoke and is best treated with bronchodilators and the removal of risk factors. The most important difference between asthma and COPD is the nature of the inflammation, which is primarily eosinophilic and CD4-driven in asthma, and neutrophilic and CD8-driven in COPD [63].

Asthma and COPD patients are mainly treated with inhalation medication, but it is difficult for many patients to use such medication correctly [64]. Thus, PMC for asthma/COPD should include education on how to use inhalation devices correctly. Furthermore, although exacerbations are potentially preventable by appropriate pharmacotherapy, patients often have difficulty following prescribed regimens. Thus, PMC for asthma/COPD may be able to enhance patient compliance and outcomes by engaging in pharmaceutical care activity such as monitoring symptoms, providing proper education on the correct handling of inhalation devices and inhalation technique, and medication counseling, helping resolve drug-related problems, and facilitating communication with physicians [65-68].

A large randomized controlled trial was conducted at 36 community drugstores (1113 participants) in the United States to assess the effectiveness of a pharmaceutical care program for patients with asthma or COPD [65]. Thirty-six drugstores were divided into 3 clusters: Pharmaceutical Care Program group, Peak Flow Monitoring Control group, and Usual Care Control group. The pharmaceutical care program provided pharmacists with recent patient-specific clinical data (peak expiratory flow rates, [PEFRs], emergency department visits, hospitalizations, and medication compliance), training, customized patient educational materials, and resources to facilitate program implementation. The PEFR monitoring control group received a peak flow meter, instructions about its use, and monthly calls to elicit PEFRs. However, PEFR data were not provided to the pharmacist. Patients in the usual care control group received neither peak flow meters nor instruction on their use; during monthly telephone interviews, PEFR rates were not elicited. The pharmaceutical care program increased patients’ PEFRs compared with those upon usual care, but provided little benefit compared with peak flow monitoring alone. Pharmaceutical care increased patient satisfaction but also increased the number of breathing-related emergency department or hospital visits (care-seeking behavior). These findings were interpreted as follows: implementation of the pharmacy program was poor, perhaps due to limited time or lack of incentives to use the resources provided, and resulted in limited benefits in terms of clinical endpoints [65].

On the other hand, a recent review article supported the National Heart, Lung, and Blood Institutes of the National Institute of Health Expert Panel Report 3 guidelines in recognizing pharmacists as accessible healthcare practitioners who, via patient education and medication management, may help patients with asthma attain better control of their disease state [66]. Furthermore, in a randomized, controlled, prospective clinical trial with a total of 133 COPD patients, significant improvements of COPD knowledge, medication adherence, medication beliefs, and a significant reduction in hospital admission rate were demonstrated by pharmaceutical care intervention delivered by clinical pharmacists [67]. A large-scale prospective cohort study in community pharmacies in Netherlands, in which the primary outcome was the reduction of oral high-dosage corticosteroids or antibiotics, demonstrated that community pharmacists actively providing comprehensive pharmacy care could improve effective treatment in asthma and COPD patients and thereby decrease the number of prescriptions for exacerbations in these patients [68].

A cross-sectional pre- to post-education/counseling study with 116 patients, who attended the PMC for asthma at Nagoya University Hospital, was conducted to assess the effects of the PMC on patients’ knowledge about the pathophysiology and drug treatment for asthma, inhalation skills, and clinical outcome [40]. The PMC significantly improved patients’ knowledge about the disease and its drug treatment. Furthermore, repeated counseling in the PMC improved PEFR and maintained it at a high level for over 2 years [40]. Subsequent studies demonstrated that pharmaceutical education and counseling in the PMC for asthma were effective in enhancing patients’ knowledge and adherence and increasing the PEFR of patients with any disease severity from mild to severe [69,70]. Significant positive correlations of medication adherence with inhalation technique and insights into the disease and medication were observed, but not with the state of disease control. It is suggested that patients who use an incorrect inhalation technique or have insufficient knowledge about the medication and pathophysiology have poor medication adherence [71].

### PMCs for cancer chemotherapy

Adherence to oral chemotherapy regimens maximizes their effectiveness and minimizes any potential toxicity. It is suggested that patients with life-threatening conditions such as cancer would be highly motivated to follow their treatment regimens [72], but the available evidence suggests that a substantial population of patients, for example, teenage and young adult patients with cancer, have difficulty adhering to treatment regimens. Factors that affect treatment adherence in young patients with cancer include patient emotional functioning (depression and self-esteem), patient health beliefs (perceived illness severity and vulnerability), and family environment [73]. A recent study in postoperative breast cancer patients before pharmacist counseling for adjuvant systemic therapy at Nagumo Clinic in Tokyo revealed that fatigue, emotional functioning, systemic therapy side effects, future perspectives, and appetite loss are determinants affecting the quality of life. Taking these factors into consideration will aid pharmacist education and counseling [74]. Treatment-related factors that may influence medication adherence include complexity of the regimen, the cost of therapy, the possibility of side effects, and the delay in treatment benefits. Meanwhile, patients may not have an adequate support system or an understanding of the need for medication, and providers may not fully succeed in communicating the importance of adherence and the types of side effects that may occur [75].

Many studies have repeatedly and consistently reported the beneficial role of clinical pharmacists and PMCs for cancer chemotherapy in oncology settings in the United States and other countries [21,22,76-82]. For instance, a cross-sectional survey revealed that patients are interested in visiting a pharmacist regularly during chemotherapy treatment and may be willing to pay for a pharmacy counseling service [21]. A retrospective analysis of clinical interventions, cost-savings, and feedback from patients and colleagues confirmed the beneficial nature of services provided by clinical oncology pharmacists in an outpatient oncology center [22]. In a recent retrospective observational cohort study assessing the effectiveness of an oral chemotherapy management (OCM) clinic providing comprehensive medication therapy management (MTM) services, including education on various oral chemotherapy agents, concurrent medications, and symptom management as well as insurance assistance, it was indicated that this clinic is effective in delivering early interventions, resulting in decreased rates of adverse effects, nonadherence, drug interactions, and medication errors over time [78].

Chemotherapy-induced nausea and vomiting (CINV) represent significant side effects in cancer patients. Poorly controlled CINV can lead to a decrease in the quality of life of patients, and to a change in the chemotherapy regimen. A 4-month longitudinal prospective intervention study was conducted to analyze the effects of pharmaceutical care (reviewing the antiemetic protocol and giving some recommendations to patients) on the incidence of delayed CINV in adult cancer outpatients. It was concluded that pharmaceutical intervention by pharmacists reduces the incidence of delayed CINV and improves medication adherence [79]. Another report described a collaborative practice agreement between pharmacists and physicians as one approach to managing chemotherapy-induced anemia in hematology-oncology patients in an anemia clinic. The pharmacist's role was justified in this clinic model through increased adherence to evidence-based practice guidelines and decreased costs associated with erythropoietin-stimulating agent therapy [83]. Alternatively, it was indicated that health-system pharmacists played an important role in screening patients with a history of breast or prostate cancer for bone loss or osteoporosis, making drug therapy recommendations to address the problem, and counseling patients on modifiable risk factors for osteoporosis and proper use of drug therapies to improve bone health [84].

In Japan, hospital pharmacists mix anticancer agents for intravenous cancer chemotherapy for both outpatients and inpatients and check the regimens. Furthermore, clinical pharmacists are expected to participate actively in cancer chemotherapy with pharmaceutical knowledge and skills to enhance patient understanding and adherence to the regimens and to secure the safety of treatment. Accordingly, the number of PMCs for cancer chemotherapy has rapidly increased in parallel with a paradigm shift of treatment from an inpatient to an outpatient basis [45-50].

The initial PMC for cancer chemotherapy was focused on patients taking TS-1 at Kyoto University Hospital, and participated in safe and effective chemotherapy for outpatients [45]. The usefulness of PMC for XELOX (capecitabine plus oxaliplatin) therapy in colorectal cancer patients was evaluated at the Cancer Institute Hospital of the Japanese Foundation for Cancer Research, in which pharmacists interviewed patients about their medication compliance, adverse effects, and supportive care, in advance of a physician’s assessments [48]. The endpoints included the number and content of inquiries about XELOX and the rate of adoption of suggestions by pharmacists for supportive care. The number of inquiries concerning XELOX was significantly fewer in the PMC group with pharmaceutical interventions (14/260 patients, 5.3%) than in the control group without consultation in the PMC (43/256 patients, 16.8%) [48]. A recent assessment of a PMC for cancer chemotherapy at the International University of Health and Welfare Mita Hospital indicated that the respective degrees of understanding of drugs, treatment schedule, and the side effects and their prevention were significantly improved by consultation in the PMC compared with those without PMC consultation. In this study, a majority of patients (44/50, 88%) who had had some anxiety about chemotherapy reported that their anxiety levels were reduced by the counseling in the PMC [49]. The outcome analysis of pharmaceutical care in outpatients who had received cancer chemotherapy at Shiga University of Medical Science Hospital indicated that the rate of amelioration of symptoms associated with chemotherapy was significantly higher (94/145 cases, 65%) when a pharmacist’s suggestions about the prescription were adopted by the physician than the rate when the suggestions were not adopted (6/17 cases, 35%) [50]. These results support the clinical significance of PMCs for cancer chemotherapy, while the cost-effectiveness of this clinical practice by pharmacists should be demonstrated in future studies.

### Future perspectives

PMCs for patient education and counseling in Japan are now expanding from anticoagulation therapy to cancer chemotherapy at hospital pharmacies. However, the contents of PMC as well as the clinical effects may vary depending upon the hospitals where the service is provided, the diseases/symptoms and pharmacotherapy that are targeted, and the knowledge and skills of individual pharmacists who participate in PMCs. The effects of PMCs on cost-effectiveness, patients’ adherence to and knowledge about pharmacotherapy, and clinical outcome have not yet been completely clarified in Japan. Furthermore, patient education and counseling at community pharmacies in the country have been limited, and there are few published reports about the implementation of PMCs at community pharmacies and their clinical impact. Last but not least, there has been almost no outreach activity for PMCs offered in Japan.

In spite of this background, PMCs for cancer chemotherapy have been approved as a new medical service in hospitals, and are covered by the universal health coverage in Japan. One might interpret this as a result of our continuous efforts; hospital pharmacists have provided pharmaceutical care and management for inpatients during the last 20 years, so their roles in pharmacotherapy management and patient education and counseling are now well recognized by medical staff and health insurance providers in Japan.

To maintain or even increase the reliability of both hospital pharmacists and community pharmacists as professionals for pharmaceutical care and management, we need to understand the current status and problems of PMCs mentioned above, and then refine the system (Table 2). There is an urgent need to develop guidelines for PMCs, which could be universally adapted in various clinical settings from cancer chemotherapy at hospital pharmacies to anticoagulation therapy and asthma/COPD treatment at community pharmacies. Such PMC guidelines should include the purpose, background, required knowledge and skills of pharmacists, the environment, the pharmacists’ roles, the steps in the patient education and counseling process, content, and documentation [1]. It is desirable for the Japanese Society of Hospital Pharmacists (JSHP) to take a leadership role in developing such guidelines in collaboration with the Japan Pharmaceutical Association. In PMCs operated in accordance with such guidelines, we should evaluate our clinical practice and provide reliable evidence that patient education and counseling by pharmacists have beneficial effects on patients’ adherence to and knowledge about their pharmacotherapy as well as the clinical outcome, besides being cost-effective. In addition, we need to undertake deep consideration of the outreach activity for PMCs, through which the roles of hospital pharmacists in pharmacotherapy management in PMCs, such as for cancer chemotherapy, can be further appreciated by society.

**Table 2:** **The proposal to improve the quality of PMCs in Japan**

1.	To develop guidelines for PMCs
2.	To provide evidence that patient education and counseling in PMCs are valuable in the following viewpoints:
	a. adherence to pharmacotherapy
	b. knowledge about pharmacotherapy
	c. clinical outcome
	d. cost-effectiveness
3.	Outreach activity for PMCs

## Conclusions

Since the first PMC in Japan was established for anticoagulation therapy with warfarin at Nagoya University Hospital in 2000, various PMCs have been expanded and implemented at hospital pharmacies in the country. As a result, PMCs for cancer chemotherapy have been approved as a new medical service in hospitals in 2014, and are covered by the universal health coverage in Japan. Although beneficial effects of PMCs on cost-effectiveness, patients’ adherence to and knowledge about pharmacotherapy, and the outcome of treatment have been repeatedly reported in the United States, the evidence is limited in Japan. There is an urgent need to develop guidelines on PMCs, which could be universally adapted in various clinical settings. We should evaluate our clinical practice and provide evidence that patient education and counseling by pharmacists are clinically valuable. Furthermore, outreach activity for PMCs should be offered, through which the roles of hospital pharmacists in pharmacotherapy management in PMC will be further appreciated by society.

## Abbreviations

ADE: Adverse drug event
ASHP: American Society of Health-System Pharmacists
CINV: Chemotherapy-induced nausea and vomiting
COPD: Chronic obstructive pulmonary disease
DOCS: Donepezil outpatient consultation service
JSHP: Japanese Society of Hospital Pharmacists
JSPHCS: Japanese Society of Pharmaceutical Health Care and Sciences
MTM: Medication therapy management
OCM: Oral chemotherapy management
PEFR: Peak expiratory flow rate
PMC: Pharmacist-managed clinic
PT-INR: Prothrombin time-international normalized ratio
